# Comparative Evaluation of DNA Extraction Methods from Feces of Multiple Host Species for Downstream Next-Generation Sequencing

**DOI:** 10.1371/journal.pone.0143334

**Published:** 2015-11-24

**Authors:** Marcia L. Hart, Alexandra Meyer, Philip J. Johnson, Aaron C. Ericsson

**Affiliations:** 1 Comparative Medicine Program, Department of Veterinary Pathobiology, University of Missouri, Columbia, Missouri, United States of America; 2 College of Veterinary Medicine, University of Missouri, Columbia, Missouri, United States of America; 3 Department of Veterinary Medicine and Surgery, University of Missouri, Columbia, Missouri, United States of America; 4 University of Missouri Metagenomics Center, University of Missouri, Columbia, Missouri, United States of America; 5 Mutant Mouse Resource and Research Center, University of Missouri, Columbia, Missouri, United States of America; University of York, UNITED KINGDOM

## Abstract

The gastrointestinal tract contains a vast community of microbes that to this day remain largely unculturable, making studies in this area challenging. With the newly affordable advanced sequencing technology, important breakthroughs in this exciting field are now possible. However, standardized methods of sample collection, handling, and DNA extraction have yet to be determined. To help address this, we investigated the use of 5 common DNA extraction methods on fecal samples from 5 different species. Our data show that the method of DNA extraction impacts DNA concentration and purity, successful NGS amplification, and influences microbial communities seen in NGS output dependent on the species of fecal sample and the DNA extraction method used. These data highlight the importance of careful consideration of DNA extraction method used when designing and interpreting data from cross species studies.

## Introduction

The gastrointestinal (GI) tract contains a vast community of microbes that greatly outnumber host cells [1] and play an important role in GI physiology [2,3], immunity of the host [4,5], and susceptibility to both local and systemic disease [6–8]. However, to date only a small percentage of these microbes can be cultured from GI biopsy tissue or fecal samples [9]. The development of culture-independent methods such as next generation sequencing (NGS) has made genomic characterization of these microbial communities increasingly feasible and affordable.

Several procedures for extraction of DNA from fecal samples have been described. Most of these studies focus on DNA extraction from human samples, with only a few of these methods reported for use on other species [10–12]. Furthermore, several studies have demonstrated that the method of DNA extraction can have an adverse impact on sequencing output and may over- or under-represent specific microbial populations in samples from different habitats [10,13–17]. Moreover, feces contain various PCR inhibitors, some of which may significantly inhibit the PCR reaction used to generate 16S rRNA amplicons prior to sequencing. The nature of these inhibitory substances in fecal samples is variable and kit-based DNA extraction methods may or may not obviate their presence. Considering the increased application of NGS to samples obtained from host species other than human and mouse, there is the need for a comparative assessment of extraction methods applied to fecal samples collected from multiple species, with 16S rRNA amplicon sequencing as the downstream application.

In this study, fresh fecal samples were collected from eight individuals within five species groups (zebrafish, mouse, cat, dog, and horse) and DNA was extracted using five different DNA extraction methods (Qiagen DNeasy kit, MoBio PowerFecal kit, Qiagen QIAamp Cador Pathogen mini kit, Qiagen QIAamp DNA Stool mini kit, and an isopropanol manual extraction method). The present study evaluates DNA extraction performance of these methods with a focus on DNA yield, purity, NGS output, and cost. Our results demonstrate that certain extraction methods, when performed according to the manufacturer’s instructions, perform preferably in samples from specific host species. Additionally, spectrophotometric assessment of DNA elutions prior to PCR had poor predictive value with regard to successful amplification and sequencing. Lastly, comparison of sequencing results from the same sample subjected to different extraction methods show that, while the detection of a limited number of rare taxa may vary, overall community profiles agreed fairly well in the majority of samples. These findings will enable investigators to choose the optimal DNA extraction method to suit current study needs.

## Materials and Methods

### Ethics Statement

All studies were performed in accordance with the recommendations put forth in the Guide for the Care and Use of Laboratory Animals and were approved by the University of Missouri Institutional Animal Care and Use Committee. For public owned animals (dog and cat) prior consent of the owner was obtained before fecal samples were obtained.

### Sample Collection

Zebrafish samples: Zebrafish (*Danio rerio*) were subjected to euthanasia with 0.1% clove oil in aquarium water, and the entire GI tract was removed and placed in a sterile 2 mL cryovial. The tissue and fecal contents were promptly frozen and stored in a -80°C freezer for two weeks prior to DNA extraction. Mouse (*Mus musculus*) samples: five freshly evacuated fecal pellets per mouse were placed in a 1.5 mL sterile Eppendorf tube. Samples were promptly frozen and stored in a -80°C freezer for two weeks. Prior to each extraction method, one fecal pellet per mouse was removed from the freezer and extraction was performed as described below. Cat (*Felis domesticus*), dog (*Canis familiaris*), and horse (*Equus caballus*) samples: freshly evacuated fecal samples were collected from privately owned cats and dogs. Freshly evacuated equine fecal samples were obtained from horses present in the University of Missouri Veterinary Medical Teaching Hospital for reasons unrelated to gastrointestinal conditions. Upon collection, samples were placed in 50 mL conical tubes and stored in a -80°C freezer for two weeks prior to DNA extraction. A small piece of fecal material was promptly removed from the frozen sample immediately prior to performance of each extraction method; the remaining sample was returned to the freezer.

### DNA extraction

All DNA was extracted and quantified at the University of Missouri Metagenomics Center (MUMC).

### Qiagen DNeasy kit

DNA extraction was performed following the manufacturer’s recommendations with slight modifications to allow for mechanical disruption. Briefly, for cat, dog, and horse samples, 25 mg of feces was placed into a sterile 2 mL round-bottom tube containing 500 μL sterile PBS and a 0.5 cm diameter stainless steel bead. Samples were mechanically disrupted using a TissueLyser II (Qiagen, Venlo, Netherlands) for 3 minutes at 30 Hz, followed by centrifugation at 200 × g for 5 min. A 200 μL volume of supernatant was removed and placed in a sterile 1.5 mL Eppendorf tube. An equal volume of buffer AL was added and the samples were processed thereafter following the manufacturer’s protocol. For mouse samples, one fecal pellet was placed into a 2 mL sterile round bottom tube containing 500 μL sterile PBS and a 0.5 cm diameter stainless steel bead and processed as described above. For fish samples, the entire GI tract was placed into an autoclaved 2 mL round-bottom tube containing 500 μL sterile PBS and a 0.5 cm diameter stainless steel bead and processed as described above. All samples were eluted in 200 μL AE buffer.

### MoBio PowerFecal kit

DNA extraction was performed following the manufacturer’s recommendations with small adaptions due to equipment availability. Briefly, for cat, dog, and horse samples, 0.25 mg of fecal sample was added to the dry bead tube containing 750 μL of bead solution and gently vortexed. C1 solution was added, the sample briefly vortexed, and incubated at 65°C for 10 minutes following the recommended protocol. Samples were shaken for 10 min. in a TissueLyser II (Qiagen, Venlo, Netherlands) at 30Hz. Samples were centrifuged at 13,000 × g for 1 min., the supernatant transferred to the provided 2 mL collection tube, and the remainder of the protocol was followed as recommended by the manufacturer. For mouse samples, one fecal pellet was added to the dry bead tube containing 750 μL of bead solution and processed as described above. For fish samples, the entire GI tract was placed in the dry bead tube containing 750 μL of bead solution and processed as described above. All samples were eluted in 100 μL solution C6.

### Qiagen QIAamp Cador Pathogen Mini kit

DNA extraction was performed following the manufacturer’s recommendations with small adaptions due to equipment availability. Briefly, for cat, dog, and horse samples 25 mg of feces was placed into a 2 mL sterile round-bottom tube containing 500 μL of sterile PBS and a 0.5 cm diameter stainless steel bead. Samples were mechanically disrupted using a TissueLyser II (Qiagen, Venlo, Netherlands) for 2 minutes at 25 Hz, followed by centrifugation at 14,000 × g for 2 minutes. A 400 μL volume of supernatant was removed and placed in a sterile 2 mL screw cap tube containing 2 mg of sterile glass beads and 100 μL of lysis buffer ATL. Samples were processed on a TissueLyser II (Qiagen, Venlo, Netherlands) for 10 min. at 50 Hz and the remainder of the protocol was followed as recommended by the manufacturer. For mouse samples, one fecal pellet was placed into a 2 mL sterile round-bottom tube containing 500 μL of sterile PBS and a 0.5 cm diameter stainless steel bead and the sample was processed as described above. For fish samples, the entire GI tract was placed in a 2 mL sterile round-bottom tube containing 500 μL of sterile PBS and a 0.5 cm diameter stainless steel bead and the sample was processed as described above. All samples were eluted in 150 μL AE buffer.

### Qiagen QIAamp DNA Stool Mini Kit

DNA extraction was performed following the manufacturer’s recommendations. Briefly, for cat, dog, and horse samples, 200 mg of feces was placed in a sterile, round-bottom 2 mL tube containing 1.4 mL ASL lysis buffer and the remainder of the protocol was followed as described by the manufacturer. For mouse samples, one fecal pellet was placed into a sterile, round-bottom 2 mL tube containing 1.4 mL lysis buffer and the sample was processed as described above. For fish samples, the entire GI tract was placed in a sterile, round bottom 2 mL tube containing 1.4 mL lysis buffer and the sample was processed as described above. All samples were eluted in 200 μL AE buffer.

### Isopropanol DNA Extraction

DNA extraction was performed as previously described [18]. Briefly, for cat, dog, and horse samples, 25 mg of feces was placed into a sterile 2 mL round-bottom tube containing 800 μL lysis buffer (500 mM NaCl, 50 mM Tris-HCl pH 8.0, 50 mM EDTA, and 4% sodium dodecyl sulfate) and a 0.5 cm diameter stainless steel bead. Samples were mechanically disrupted using a TissueLyser II (Qiagen, Venlo, Netherlands) for 3 minutes at 30 Hz, followed by incubation at 70°C for 20 minutes with periodic vortexing. Samples were centrifuged at 5000 × g for 5 min., and the supernatant was then transferred to a sterile 1.5 mL Eppendorf tube containing 200 μL of 10 mM ammonium acetate. Lysates were vortexed, incubated on ice for 5 min., and then centrifuged. Supernatant was transferred to a sterile 1.5 mL Eppendorf tube and one volume of chilled isopropanol was added. Samples were incubated on ice for 30 min. and then centrifuged at 16,000 × g, at 4°C, for 15 min. The resulting DNA pellet was washed with 70% ethanol and resuspended in 150 μL Tris-EDTA (10 mM Tris and 1 mM EDTA), followed by addition of 15 μL of proteinase K and 200 μL of AL Buffer. Samples were incubated at 70°C for 10 min. and 200 μL of 100% ethanol was added to the tubes. Samples were mixed by gentle pipetting and the contents transferred to a spin column from the DNeasy kit. The DNA was purified following the manufacturer’s instructions and eluted in 200 μL EB buffer. For mouse samples, one fecal pellet was placed into a sterile 2 mL, round-bottom tube containing lysis buffer and a 0.5 cm diameter stainless steel bead and the sample was processed as described above. For fish samples, the entire GI tract was placed into a sterile 2 mL round-bottom tube containing lysis buffer and a 0.5 cm diameter stainless steel bead and the sample was processed as described above. All samples were eluted in 200 μl EB buffer.

### Quantification and assessment of purity

For all extraction methods, DNA concentrations were determined fluorometrically (Qubit dsDNA BR assay, Life Technologies, Carlsbad CA) and purity was assessed via 260/280 and 260/230 absorbance ratios, as determined via spectrophotometry (Nanodrop 1000 Spectrophotometer, Thermo Fisher Scientific, Waltham, MA). Samples were stored at -20°C until sequencing.

### Library Construction and 16S rRNA Sequencing

Library construction and sequencing was performed at the University of Missouri DNA Core facility. DNA concentration of samples was determined fluorometrically and all samples were normalized to 3.51 ng/μL for PCR amplification. Bacterial 16S rRNA amplicons were generated via amplification of the V4 hypervariable region of the 16S rRNA gene using single-indexed universal primers (U515F/806R) flanked by Illumina standard adapter sequences and the following parameters: 98°C^(3:00)^+[98°C^(0:15)^+50°C^(0:30)^+72°C^(0:30)^] × 25 cycles +72°C^(7:00)^. Amplicons were then pooled for sequencing using the Illumina MiSeq platform and V2 chemistry with 2×250 bp paired-end reads, as previously described [18]. Samples returning greater than 10,000 reads were deemed to have successful amplification.

### Informatics analysis

Assembly, binning, and annotation of DNA sequences was performed at the MU Informatics Research Core Facility. Briefly, contiguous DNA sequences were assembled using FLASH software [19], and culled if found to be short after trimming for a base quality less than 31. Qiime v1.8 [20] software was used to perform *de novo* and reference-based chimera detection and removal, and remaining contiguous sequences were assigned to operational taxonomic units (OTUs) via *de novo* OTU clustering and a criterion of 97% nucleotide identity. Taxonomy was assigned to selected OTUs using BLAST [21] against the Greengenes database [22] of 16S rRNA sequences and taxonomy. Principal component analyses were performed using ¼ root-transformed OTU relative abundance data via a non-linear iterative partial least squares (NIPALS) algorithm, using an open access Excel macro available from the Riken Institute (http://prime.psc.riken.jp/Metabolomics_Software/StatisticalAnalysisOnMicrosoftExcel/index.html).

### Statistical analysis

Statistical analysis was performed using Sigma Plot 12.3 (Systat Software Inc., Carlsbad, CA). Differences between DNA extraction methods in total DNA concentration were determined using ANOVA with Student Newman-Keuls post hoc test. Differences in phylum relative abundance (non-rarefied) following successful NGS amplification, i.e., achieving 10,000 sequences, were determined using either ANOVA with Student Newman-Keuls post hoc test or a t-test with Mann-Whitney post hoc test. Receiver operating characteristic (ROC) curves were generated from 260/280 and 260/230 absorbance ratios obtained from samples of each host species using success of amplification as the binary classifier. Statistical differences in microbial diversity were determined at a uniform depth using ANOVA with Student Newman-Keuls post hoc test. Results were considered statistically significant for *p* values ≤ 0.05.

## Results

### DNA extraction method impacts DNA yield

**Fig 1:**
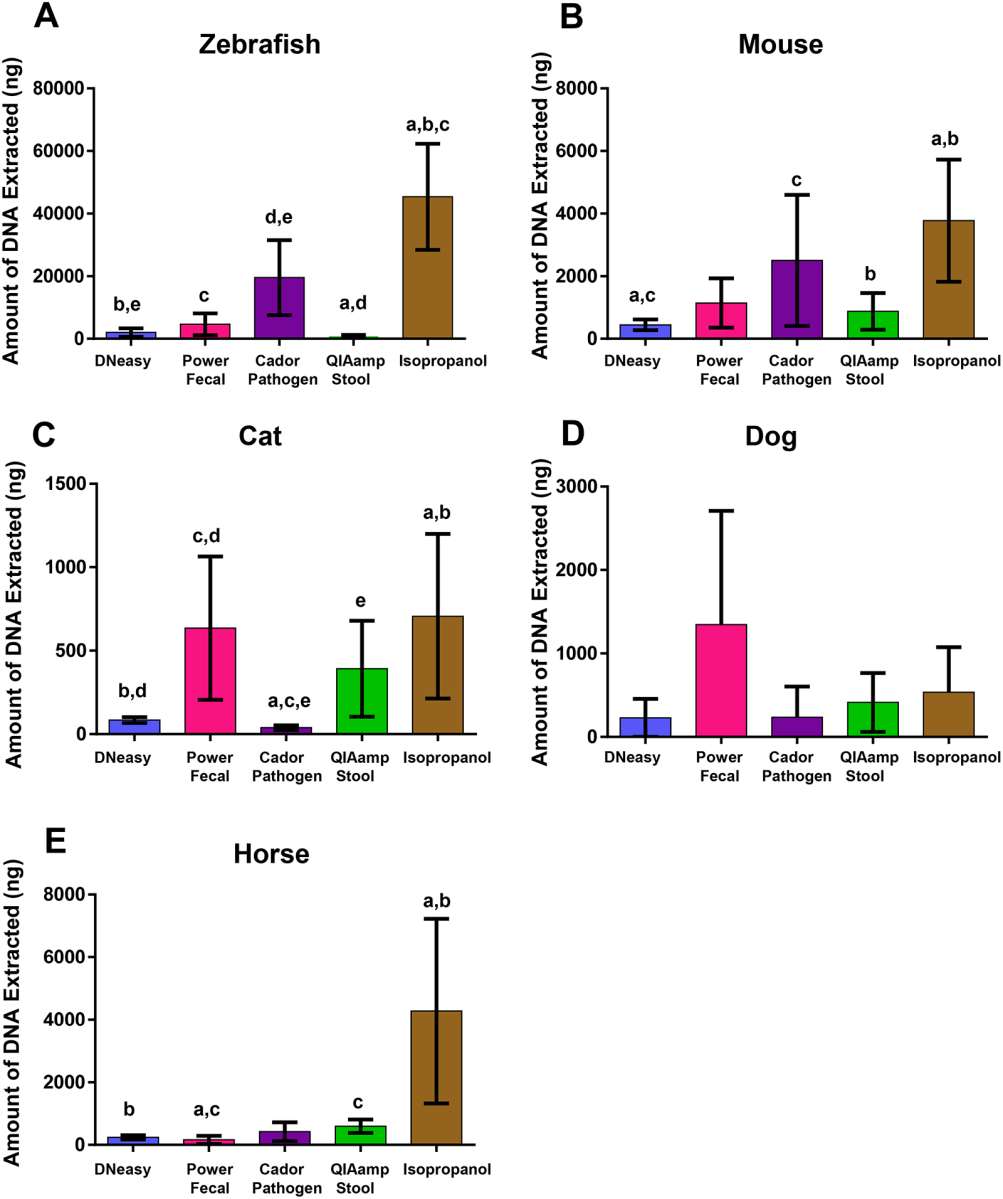
Fecal DNA extraction efficiency varies dependent on extraction method and host species. Mean total amount (± standard deviation) of DNA extracted from zebrafish gastrointestinal tract (A) or a standardized mass of feces from mice (B), cats (C), dogs (D), or horses (E), using four commercially available DNA extraction kits and one manual extraction procedure (isopropanol). *n* = 40 individual zebra fish, and 8 individuals for mouse, cat, dog, and horse with 8 samples used for all extraction methods. Samples were extracted and total DNA was measured by fluorometry. Statistical significance determined using one way ANOVA with Student Newman-Keuls post hoc test. Significance defined by *p*≤0.05 and denoted by like lower case letters, i.e., samples marked with the same letter are significantly different. Whiskers denoting lower standard deviation are cropped at zero.

In addition to traditional “bench-top” techniques for DNA extraction, there are many commercially available kit-based methods. To assess the suitability of several of these methods for extraction of fecal DNA from multiple host species with the intended downstream use of next-generation sequencing, samples were collected from eight individual mice, cats, dogs, horses, or forty zebrafish, and processed according to the manufacturer’s protocol or published methods. For cat, dog, and horse samples, the same fecal bolus was used for all extraction methods. For mice, five fecal samples were collected at the same time and one pellet was used for each method. For zebrafish, forty age-matched fish from the same tank were used, eight per extraction method. Regarding overall DNA yield from a standard mass of starting material, the amount of DNA extracted was dependent on the DNA extraction technique and the host species from which the fecal sample was collected. For all species examined, except the dog, the isopropanol extraction method produced the greatest total DNA yields, and in horse samples, the isopropanol method resulted in significantly higher yields when compared to all other extraction methods (Fig 1A–1E). In the dog, no statistically significant difference between extraction methods was identified (Fig 1D). For zebrafish and mouse samples, the isopropanol extraction method resulted in significantly greater yields of total DNA relative to several other methods, with the Cador Pathogen kit also performing well (Fig 1A–1B). For cat samples, elutions from the isopropanol extraction method were significantly higher than both the Cador Pathogen and DNeasy kit, with comparable yields from the PowerFecal and QIAamp Stool kits (Fig 1C). While a greater DNA yield may suggest, among other things, more efficient bacterial lysis, DNA is normalized to a standard volume and concentration for 16S rRNA amplification. With that in mind, most methods examined in this study provided a sufficient quantity of DNA for normalization and attempted sequencing with the exceptions of DNeasy and PowerFecal extractions from equine samples, DNeasy and Cador Pathogen extractions from feline samples, and QIAamp stool extractions from zebrafish samples.

### DNA extraction method influences successful amplification and next generation sequencing (NGS)

A more practical measure of performance is the quality of the DNA elutions generated from each method. The presence of myriad undesirable substances in feces is often blamed for samples that result in low coverage (i.e., sequences per sample) due to PCR inhibition. To assess the quality of the DNA extracted via the methods under evaluation, samples were amplified in a 96-well format using single-indexed primers, and pooled for sequencing on the Illumina MiSeq platform. Based on rarefaction analysis of previously published sequencing data [18], we defined successful amplification as a minimal coverage of 10,000 reads per sample. Notably, the method of DNA extraction had a strong impact on the number of samples that successfully amplified, but performance varied between host species. For example, with zebrafish samples, the isopropanol extraction method was most efficient at overcoming PCR inhibition; 8/8 isopropanol-extracted zebrafish samples amplified successfully, compared to between 0/8 and 4/8 samples using DNA extracted via the other methods tested (Table 1). Similarly, the PowerFecal kit appeared to be optimal for extraction of DNA from equine feces (7/8 samples amplified); 5/8 samples extracted with the QIAamp Stool kit amplified successfully. For mouse samples, the PowerFecal-, QIAamp stool-, and isopropanol-extracted samples all performed well with all 8/8 samples amplifying above 10,000 reads. For cat and dog samples, the PowerFecal kit was most able to overcome PCR inhibition, resulting in successful amplification in 7/8 and 8/8 samples, respectively. Isopropanol-extracted dog samples performed well in 7/8 samples. Overall, the PowerFecal kit and the traditional isopropanol extraction method provided the most consistently successful PCR amplification prior to sequencing bacterial DNA from diverse host species, when samples were handled according to the manufacturer’s recommendations. While murine feces seemed the least problematic with regard to PCR inhibition, zebrafish, cat, and horse fecal samples each required a specific extraction method for optimal results.

**Table 1:** Comparison of DNA extraction methods. Cost of DNA extraction methods calculated on per sample basis to include cost of kits, Eppendorf tubes, stainless steel beads, and pipette tips. Time of extraction method determined from start of fecal processing to DNA elution. Mean 260/280 and 260/230 nm absorbance (as determined by spectrophotometry) and standard deviation for all DNA extraction methods for each animal species. Number of amplified samples determined based on the total number of samples resulting in greater than 10,000 reads. *n* = 8 per extraction method.

Extraction Method	Cost ($)	Time (hr)	Mean A260/A280 ± s.d.					Amplification				
			Mean A260/A230 ± s.d.									
			Fish	Mouse	Cat	Dog	Horse	Fish	Mouse	Cat	Dog	Horse
**DNeasy**	3.16	1–1.5	2.0 ±0.02	2.0 ±0.23	1.83 ±0.15	2.13 ±0.49	1.56 ±0.19	4/8	3/8	5/8	5/8	0/8
			2.06 ±0.13	0.95 ±0.29	0.26 ±0.04	0.39 ±0.18	0.53 ±0.06					
**PowerFecal**	5.26	1.5–2	1.85 ±0.09	1.9 ±0.23	1.57 ±0.19	1.65 ±0.18	1.66 ±0.21	3/8	8/8	7/8	8/8	7/8
			2.02 ±0.40	1.14 ±0.36	1.0 ±0.27	1.31 ±0.60	0.38 ±0.04					
**Cador**	4.06	1.5–2	2.0 ±0.04	1.9 ±0.23	3.11 ±2.22	2.19 ±0.19	2.46 ±0.94	0/8	3/8	1/8	5/8	2/8
			1.78 ±0.32	1.7 ±0.38	0.50 ±0.31	0.64 ±0.49	0.34 ±0.1					
**QIAamp**	4.32	1.5–2	2.1 ±0.05	2.2 ±0.62	2.07 ±0.07	2.13 ±0.13	3.32 ±0.72	0/8	8/8	1/8	2/8	5/8
			2.19 ±1.06	2.03 ±1.06	0.82 ±0.48	1.67 ±0.41	0.07 ±0.01					
**Isopropanol**	3.51	3.5–4	1.98 ±0.04	1.9 ±0.06	2.38 ±0.62	2.2 ±0.31	1.84 ±0.45	8/8	8/8	5/8	7/8	1/8
			2.18 ±0.12	1.9 ±0.23	0.82 ±0.48	0.85 ±0.32	0.72 ±0.57					

### DNA purity does not predict successful amplification and sequencing

**Fig 2:**
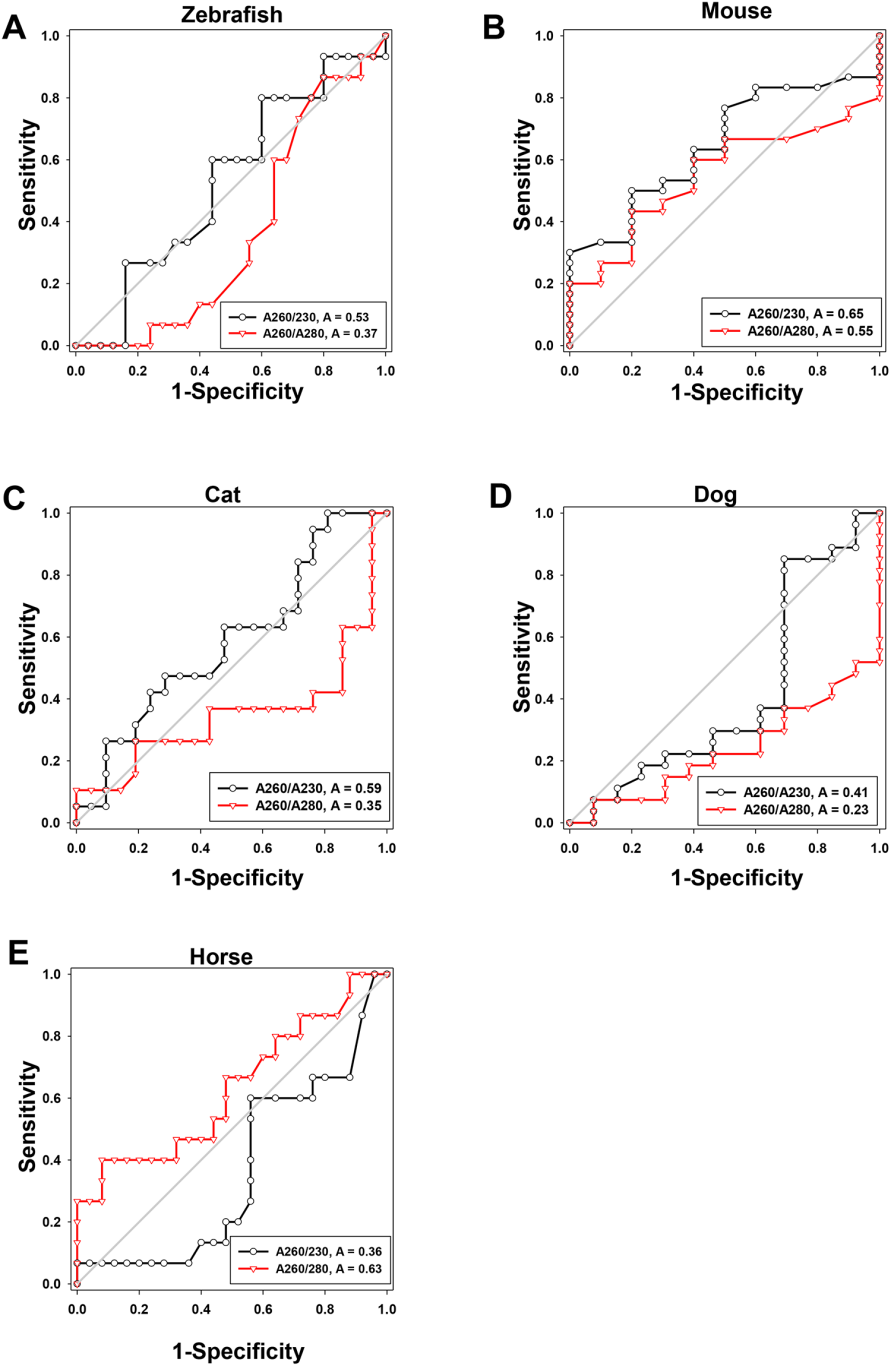
Receiver operator curves of 260/280 and 260/230 nm absorbance for all DNA extraction methods for each animal species. Absorbance values of all DNA samples from each species (*n* = 40) were plotted in an ROC generated by Sigma-Plot.

Prior to NGS, successful PCR-based amplification requires preparation of template DNA containing little to no protein, RNA, or polysaccharides, and purity is often assessed via 260/280 and 260/230 absorbance ratios, determined by spectrophotometry. To compare the predictive power for successful amplification of each absorbance ratio, and also to establish cut-off values for one or both of the ratios, receiver-operating characteristic (ROC) curves were generated for each species, with successful amplification as the binary classifier. ROC curves are frequently used in the development of diagnostic assays to compare positive and negative predictive values of test results, and to determine a threshold value with acceptable sensitivity and specificity based on test results of a group of “known” samples. The area under the curve (AUC) can be used as a measure of overall diagnostic (or in this case, predictive) accuracy; an AUC of 1 denotes 100% sensitivity and specificity while an AUC of 0.5 suggests completely random performance. Unexpectedly, neither high 260/280 or 260/230 absorbance ratios accurately predicted the success of NGS amplification (Fig 2 and Table 1). For example, DNA from fish samples produced excellent absorbance ratios suggesting relatively pure DNA, but four of five extraction methods either failed to have any successful amplification or had few samples amplify. Conversely, in cat, dog, and horse samples, several DNA extraction methods resulted in extremely low 260/230 absorbance ratios yet produced samples that amplified and sequenced well.

### Effect of DNA extraction method on microbial diversity is dependent on host species

**Fig 3:**
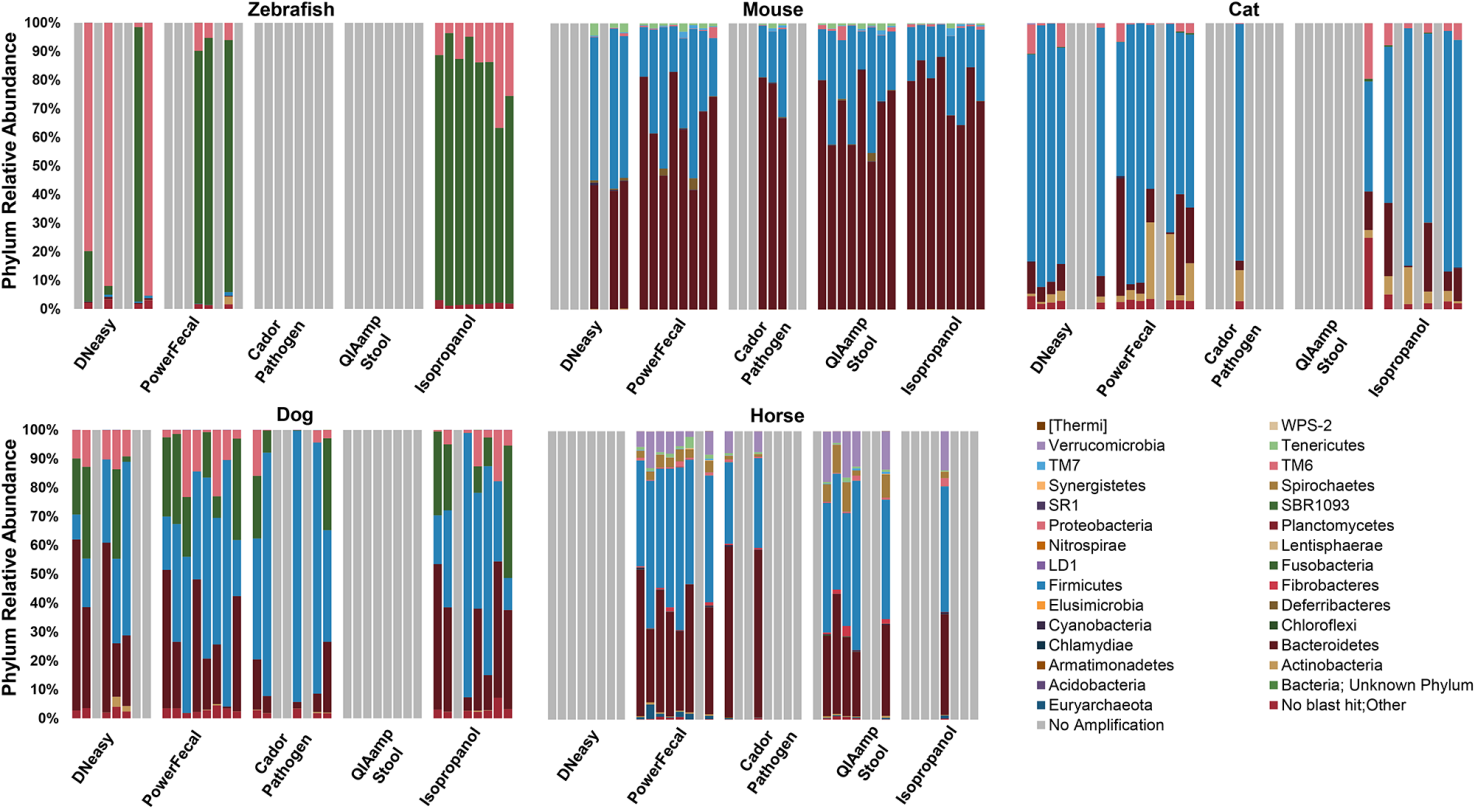
Comparison of DNA extraction method on next generation sequencing (NGS) relative abundance at the phylum level. Gray bars represent samples that resulted in sequencing below 10,000 reads. *n* = 8 samples per extraction method.

**Fig 4:**
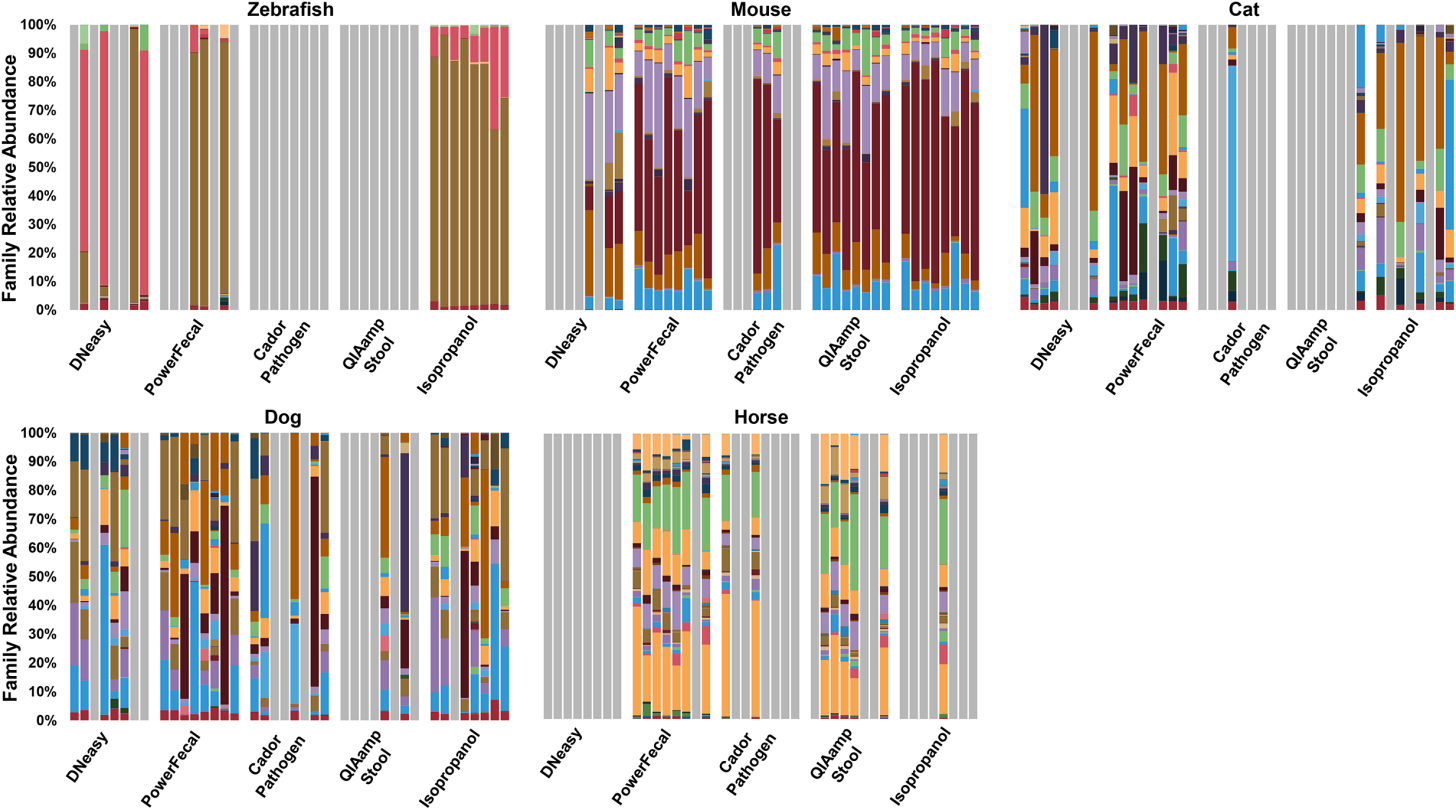
Comparison of DNA extraction method on next generation sequencing (NGS) relative abundance at the family level. Gray bars represent samples that resulted in sequencing below 10,000 reads. *n* = 8 samples per extraction method.

In human studies, it has been shown that variation in DNA extraction can influence the bacterial communities detected during sequencing (Kennedy et al. 2014; Wesolowska-Anderson et al. 2014; Wu et al. 2010). To determine whether the extraction chemistries tested resulted in differential lysis and subsequent skewing of the microbial profile, results of 16S rRNA amplicon sequencing were compared. Annotated to the taxonomic level of phylum, those samples that reached the threshold of 10,000 sequences generally showed good agreement with some exceptions. For example, sequencing of zebrafish samples extracted using the PowerFecal kit or manual isopropanol method detected a higher relative abundance of microbial families within the phylum *Fusobacteria* (Figs 3 and 4) when compared to samples processed with the DNeasy kit. Specifically, three of the four zebrafish samples processed with the DNeasy kit were dominated by greater than 80% *Proteobacteria*, while the predicted relative abundance of that phylum was less than 40% in the isopropanol extracted samples. The fact that zebrafish samples represented 40 individual fish, as opposed to 8 samples divided into 5 subsamples precluded statistical comparisons. Sequencing of samples from the other species demonstrated relatively lesser differences at the phylum level, although certain samples yielded disparate results. However, when annotated to the level of family, the microbial profile detected in cat and dog samples demonstrated clear differences between extraction methods in samples that amplified (Fig 4). In the mouse and horse, all DNA extraction methods resulted in subjectively similar relative abundance detected at the family levels. Based on the likelihood of success of amplification, the PowerFecal kit appears optimal for extraction of cat and horse samples, while the manual isopropanol extraction method performed best for zebrafish samples. With dog and mouse samples however, there were two or three methods, respectively, that successfully overcame PCR inhibition in seven or more of the eight samples tested, allowing for statistical comparisons of the output. Accordingly, a t-test performed on the relative abundance of all phyla detected in the seven dog samples extracted with the PowerFecal kit or isopropanol method revealed no significant differences. Similarly, an ANOVA was performed to test for differences in the relative abundance of all phyla detected in the eight mouse samples extracted with the PowerFecal kit, QIAamp kit, or isopropanol method. A significant difference (*p* = 0.035) was detected in the relative abundance of the rare phylum Cyanobacteria, with QIAamp-processed samples showing greater abundance when compared to isopropanol-extracted samples. No differences were detected in the relative abundance of any other phylum in the mouse samples extracted with those three methods.

**Fig 5:**
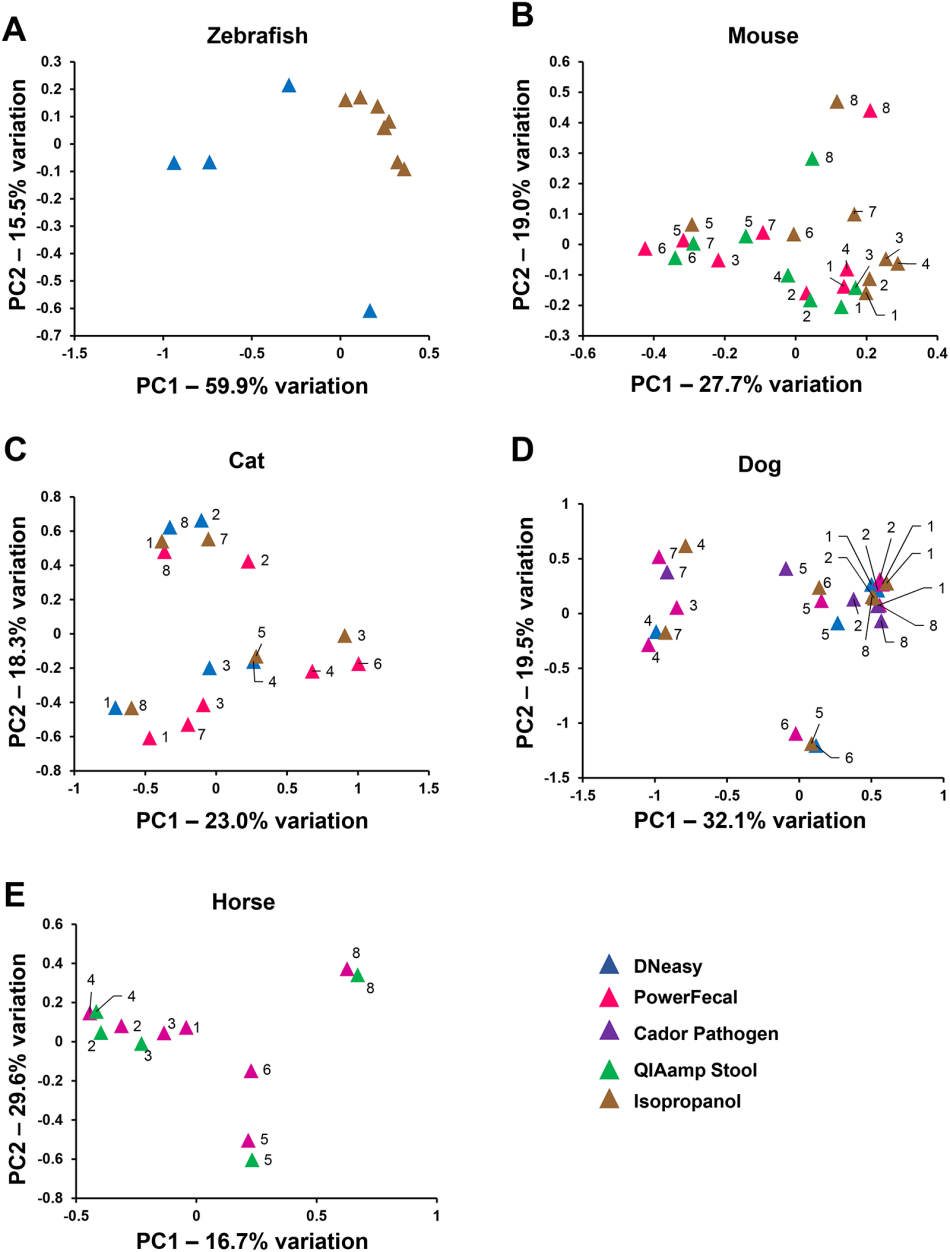
Principal Component Analysis (PCA) of samples with successful amplification and sequencing in at least half (4/8) samples. Colors denote extraction method: DNeasy (blue), PowerFecal (pink), CadorPathogen (purple), QIAmp Stool (green), and Isopropanol (brown). Numbers denote individual animal samples tracked across all kits tested for that species

**Fig 6:**
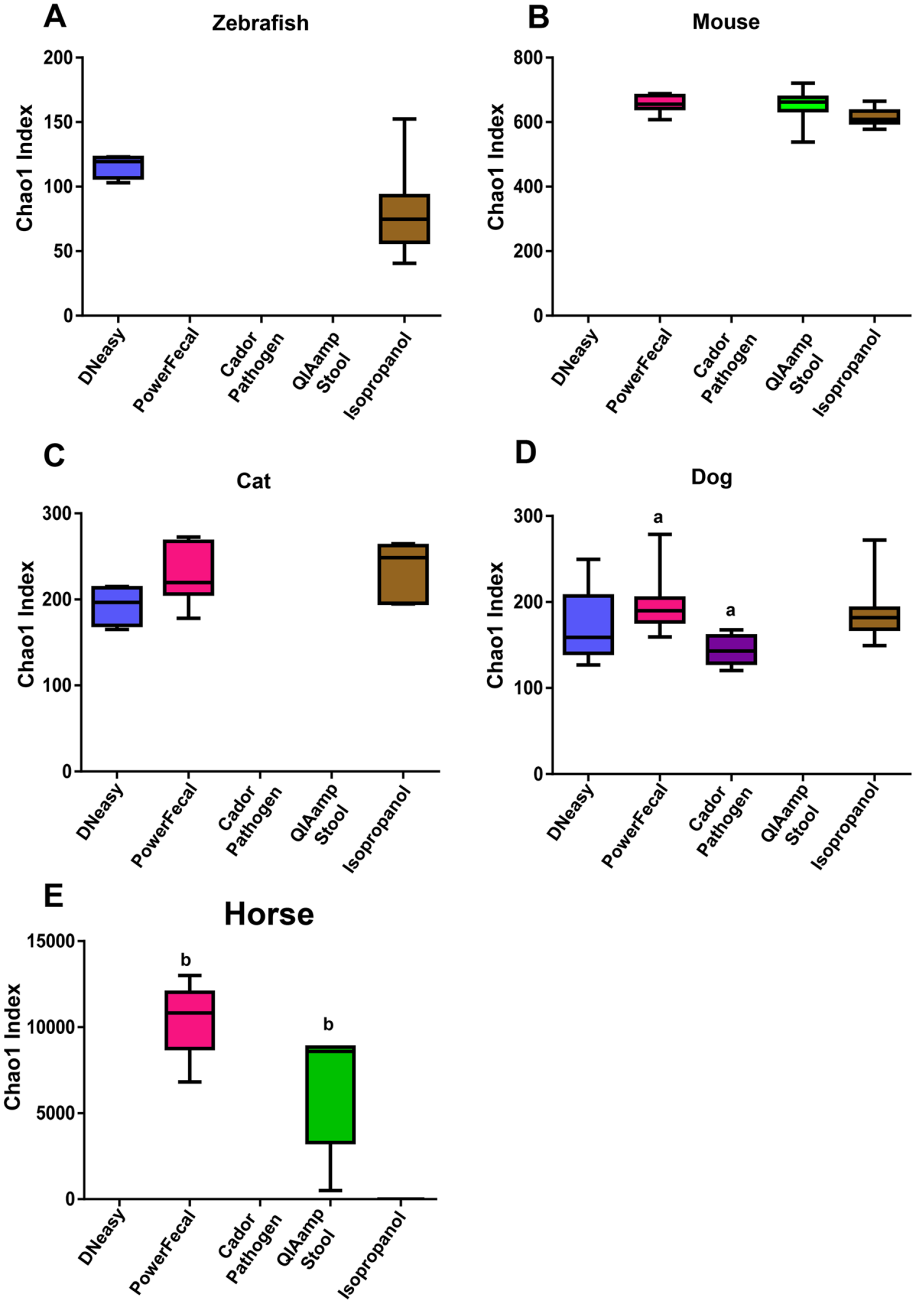
Diversity of fecal microbiota. Chao1 estimate of microbial diversity plotted by Tukey box and whisker graph. For zebrafish and horse samples, statistical significance was determined using student’s t-test. For mouse, cat, and dog samples, statistical significance was determined using ANOVA with Student Newman Keuls post hoc test. Statistical significance defined by *p*≤0.05 and denoted in the figure by lower case letters.

To further evaluate the effect of DNA extraction method on NGS output, principle component analysis (PCA) was performed including only data from methods that resulted in successful amplification in half or more samples for each host species (Fig 5). Data from methods that resulted in successful amplification in less than half of the samples were omitted from PCA to avoid skewing of comparisons between the other methods. In PCA, samples that are similar in composition cluster together, based on the presence or absence and relative abundance of all operational taxonomic units (OTUs), i.e., taxa annotated to the finest resolution possible with the primer set being used. As expected based on the bar charts (Fig 4), the variation among zebrafish samples extracted using the isopropanol method was much lower than samples isolated using the DNeasy kit (Fig 5A). In the mouse and horse, individual animal samples tended to cluster together regardless of which DNA extraction method was used (Fig 5B and 5E). In the dog and cat, individual animal samples clustered along the PC1 axis. However, dependent on which DNA extraction method was used, several samples separated along PC2. As a means of comparing the ability of tested extraction methods to lyse rare or hard-to-lyse taxa, α-diversity of samples that amplified was determined using the Chao1 Index (Fig 6). Despite the paucity of significant differences detected between methods, a few trends were noted. In mouse samples, microbial diversity was highly concordant across all extraction methods capable of overcoming PCR inhibition. In the dog samples, there was considerable variability in the Chao1 indices of samples generated via most extraction methods, perhaps reflecting an increased exposure to environmental microbes, or differences between portions of the same fecal sample. Interestingly, equine samples had statistically greater microbial diversity in samples extracted using the PowerFecal kit, relative to those QIAamp Stool-extracted samples that amplified successfully. Collectively, these data illustrate that successful amplification and sequencing, as well as the microbial profile detected via NGS, can be influenced by DNA extraction method and, depending on host species and the output of interest, these changes may be dramatic or subtle.

## Discussion

With the decrease in costs associated with NGS, characterization of GI microbial communities is becoming increasingly feasible. However, to date, the majority of studies have focused on human fecal samples with far less optimization of techniques for use with samples from other species. In the current study, the performance of five DNA extraction methods was evaluated using samples collected from five diverse host species. Taken as a whole, the data suggest that samples from a particular host species may be amenable to DNA extraction using only certain methods.

This phenomenon was first noticed when comparing the concentration of DNA in elutions from each method. While the manual isopropanol precipitation yielded consistently high levels of DNA, the PowerFecal kit resulted in comparable yields from zebrafish and mouse samples, and the DNeasy kit produced comparable yields in feline and canine samples. One possible explanation for the greater yields generated via isopropanol extraction is the fact that the other kit-based methods rely solely on retention of DNA in a column-based matrix. In the isopropanol method, DNA is precipitated via addition of a chaotropic salt and isopropanol on ice, followed by subsequent elution over a column. That said, other differences in the extraction chemistry may also influence efficiency of lysis and removal of PCR inhibitors.

We also noted considerable variability in the amount of DNA extracted within several samples, dependent on extraction method used, as evidenced by the large standard deviation among sample groups. This variation could be due to the efficiency of the DNA extraction methods tested. However, this could also be due to variability in the diet that these animals consume or the amount of true microbial biomass loaded with each sample as cat and dog samples were found to contain a large amount of particulate matter such as hair and bone fragments. Similarly, horse fecal samples contained substantial amounts of large particulate fiber matter. Although care was taken during measurement of starting sample mass, these particulates could have an effect on the amount of fecal biomass that was available for DNA extraction.

More importantly, the method of DNA extraction was found to strongly influence the number of samples that successfully amplified, but in a species-dependent fashion. For example, the isopropanol method was uniquely well-suited for use with zebrafish samples, resulting in successful amplification in 8/8 samples, while successful amplification and sequencing of equine samples necessitated use of the PowerFecal kits. Taken together, these data suggest that the substances present in the feces responsible for PCR inhibition likely differ between host species.

Furthermore, all zebrafish samples had high A260/A280 and A260/A230 absorbance ratios, suggesting relatively “clean” elutions, but three out of the five extraction methods tested completely failed NGS amplification. This may be an artifact of processing as the entire zebrafish GI tract was processed along with fecal contents. Extracted samples likely contained a large amount of host DNA which may have resulted in dilution of bacterial DNA when performing PCR with a standard DNA concentration. However, yields from isopropanol-extracted samples were noted to be the highest and performed well. Conversely, in the cat, dog and horse samples, both high and low A260/A280 and A260/A230 absorbance values were detected, possibly indicating contamination from guanidine residue during DNA extraction or dietary carbohydrate carryover. These samples were thus expected to have variable to unsuccessful amplification, however several such samples successfully amplified. Taken together, these results indicate that DNA purity, as determined via 260/280 or 260/230 absorbance ratio, cannot reliably predict successful amplification.

Several studies have shown that variation in DNA extraction can impact NGS-based characterizations of bacterial communities. In the present study, only small differences in NGS output were observed for most samples at the phylum level, with the aforementioned exception of zebrafish samples. In zebrafish however, use of the PowerFecal kit or the isopropanol method resulted in detection of a higher relative abundance of the phylum *Fusobacteria*, while samples extracted via the DNeasy kit showed predominantly *Proteobacteria*. When overall microbial profiles were compared at the level of OTU via PCA, we noted substantial variation among the samples extracted with the DNeasy kits. Due to the scant amount of digesta present in zebrafish, DNA was extracted from individual fish as compared to serial or identical fecal samples from other species evaluated in this study. Even though all fish used for this study were specific pathogen free and housed in the same aquarium, differences in OTU variation could be due to true differences between individual fish. That said, it is worth noting that the community profiles generated using the isopropanol extraction method are not in agreement with other published reports of zebrafish microbiota [23]. While Roeselers *et al*. and other groups have identified *Proteobacteria* as the predominant phylum in the gut of zebrafish, *Fusobacteria* was the primary phylum detected in the current study. Whether this is a function of extraction method or due to a difference in the populations tested is unclear.

Collectively, these data highlight the need to match appropriate fecal DNA extraction methods to the host species in question. As the feasibility of NGS increases and with rising pressure from funding agencies to enhance experimental reproducibility [24], it is paramount that standardized methods of sample processing be performed. This study illustrates the importance of careful consideration of DNA extraction method when designing experiments and interpreting data from studies performed in multiple species.
